# Phase II Study of Gemcitabine and Docetaxel Combination in Patients with Previously Treated Recurrent or Metastatic Squamous Cell Carcinoma of the Head and Neck

**DOI:** 10.5402/2012/159568

**Published:** 2012-05-10

**Authors:** Zyad Kafri, Lance K. Heilbrun, Ammar Sukari, George Yoo, John Jacobs, Ho-Sheng Lin, Heather Mulrenan, Daryn Smith, Omer Kucuk

**Affiliations:** ^1^Barbara Ann Karmanos Cancer Institutte, Wayne State University School of Medicine, Detroit, MI 48201, USA; ^2^Division of Hematology/Oncology, St. John Hospital, Wayne State University School of Medicine, 19229 Mack Avenue, Grosse Pointe Woods, Detroit, MI 48236, USA

## Abstract

*Purpose*. To explore the safety and efficacy of gemcitabine and docetaxel (GEMDOC) in previously treated patients with recurrent or metastatic squamous cell carcinoma of the head and neck (SCCHN). *Patients and Methods*. Patients with advanced SCCHN previously pretreated with one or two lines of palliative chemotherapy were treated with gemcitabine and docetaxel until disease progression. *Results*. Thirty-six patients were enrolled, and 29 were response evaluable. 16 (55%) experienced clinical benefit (response or stable disease). Six (21%) patients achieved partial response (PR), none achieved complete response (CR), and the overall response rate (ORR) was 21% (95% CI: 0.10–0.38). Ten (28%) patients had stable disease. The median response duration (RD) for the 6 PR patients was 3.2 months (80% CI: 2.0–6.1 months). Median overall survival was 4.2 months (95% CI: 2.4–7.0 months). Among the 33 treated patients: 13 (39%) patients had grade 3-4 anemia, 10 (30%) had grade 3-4 neutropenia. *Conclusion*. The study drugs were relatively safe, and the clinical benefit (PR + SD) rate was 55%. However, the efficacy objective for this regimen was not met. Given the good safety profile, further investigation of this regimen with the addition of a targeted agent may lead to better efficacy.

## 1. Introduction

Patients with squamous cell carcinoma of head and neck (SCCHN) who had disease recurrence after primary surgery or chemoradiation therapy or who present with metastatic disease usually have a poor prognosis [1]. The role of chemotherapy in this setting is palliative, complete response is rare, and duration of response is short.

Single-agent docetaxel was previously evaluated in four phase II studies involving approximately 160 patients with metastatic or recurrent SCCHN. The overall response rates observed in these studies ranged from 21% to 42% [2–5]. The principal toxicity reported in these studies was grade 3 and 4 neutropenia. Based on these phase II studies, single-agent docetaxel 75–100 mg/m^2^ IV every 3 weeks was shown to be an active and generally well-tolerated regimen for metastatic or recurrent SCCHN.

Docetaxel has also been evaluated as part of a doublet in combination with cisplatin or 5-fluorouracil (5-FU) in patients with recurrent or metastatic SCCHN. In phase I/II trials evaluating the combination of docetaxel and cisplatin the overall response rates were 33% to 40% [6–8]. Studies evaluating the combination of docetaxel and 5-FU yielded response rates from 24% to 27% [9, 10]. Of the two doublets, docetaxel combined with cisplatin appeared to be the more effective regimen in regard to both objective and complete responses [6–9]. In a phase II study conducted by EORTC, the response rate to the docetaxel plus cisplatin arm was 86% in patients with chemotherapy and radiation naïve disease versus 33% for the pretreated group [7]. As with single-agent docetaxel, the primary toxicity in each series was myelosuppression.

Gemcitabine has shown significant activity in a wide variety of solid tumors, including cancers of pancreas [15], breast [16], lung [17], and ovary [18]. A trial of gemcitabine in head and neck cancers used a relatively low dose (800 mg/m^2^) and reported 7 partial responses (11%) in 62 patients [19]. Multiple investigators have evaluated the combination of gemcitabine and docetaxel (GEMDOC) in phase II clinical trials on biweekly bases. [11–14] Previous phase II studies of biweekly GEMDOC have included patients with non-small-cell lung cancer, breast cancer, and pancreatic cancer and are summarized in Table 1. The doses that we selected from our previous phase I/II trials experience in head and neck cancer at our facility were 3000 mg/m^2^ for gemcitabine and 60 mg/m^2^ for docetaxel.

The biweekly GEMDOC combination is an attractive regimen in the treatment of advanced head and neck cancer since both agents are active against SCCHN with a low toxicity profile. Therefore, we conducted this phase II clinical trial to explore the efficacy and toxicity of the GEMDOC combination given biweekly for patients previously treated with recurrent or metastatic SCCHN.

## 2. Patients and Methods

### 2.1. Patients

Patients with recurrent or metastatic histologically proven SCCHN who had received 1 to no more than 2 prior chemotherapy regimens were eligible. Patients who received a prior taxane agent or gemcitabine were not eligible. Patients were required to have at least one bidimensional measurable disease site, assessed by radiologic exam performed and documented within 28 days prior to registration. Additional eligibility criteria included a treatment-free interval of at least 4 weeks prior to study entry, and no CNS metastases. Patients must have had a SWOG performance status of ≤2, adequate hematologic cell counts (absolute neutrophil count ≥1,500/*μ*L and platelets ≥100,000/*μ*L), and adequate liver and renal function.

The institutional review board of the participating center approved the study, and all patients provided signed informed consent.

### 2.2. Study Design and Treatment

This was a prospective, phase II evaluation of biweekly doses of gemcitabine and docetaxel. Treatment was given as gemcitabine 3000 mg/m^2^ IV over 30 minutes followed by docetaxel 60 mg/m^2^ IV over 60 minutes. Appropriate antiemetics were used as premedication as well as dexamethasone, either 8 mg PO BID starting one day before each dose of docetaxel for 3 days or as 20 mg IV prior to docetaxel infusion [20]. Treatment was repeated every two weeks with appropriate dose modifications until disease progression, unacceptable toxicity, or complete remission plus 4 cycles, whichever occurred first. Patients continued to receive treatments in the absence of any grade 2 or higher toxicities. Infusion was given on day 1 of treatment with the appropriate dose adjustment if absolute neutrophil count was >1,000/*μ*L and platelet count was >50,000/*μ*L. Treatment delay and dose reductions for docetaxel and gemcitabine were implemented for grade 3-4 neutropenia, thrombocytopenia, or liver dysfunction. Patients who failed to achieve hematologic recovery for 3 consecutive weeks or more than 4 weeks for nonhematologic toxicities were removed from the study.

### 2.3. On-Study Evaluation

Tumor response was assessed radiographically with standard methods using RECIST criteria [21] every 4 cycles (approximately 2 months). Toxicity was evaluated before each cycle. All patients were considered (regardless of the number of cycles they received) evaluable for response.

The best overall response was the best response that was recorded from the start of treatment until disease progression. The duration of response was measured from the time measurement criteria were met for complete response (CR) or partial response (PR) (whichever status was recorded first) until the first date that recurrence or progressive disease (PD) was recorded since the treatment started. Duration of stable disease (SD) was measured from the start of treatment until the criteria for disease progression were met. Response-evaluable patients were those who were registered and had their response evaluated and determined by appropriate measurements, regardless of the number of chemotherapy cycles they received. Toxicity-evaluable patients were those who received chemotherapy or any portion of a cycle of therapy. Patients were taken off the study if they had documented PD, an unacceptable adverse event, patient decision to withdraw from the study, investigator judgment to stop treatment, or upon completion of 4 cycles after first documented CR.

The safety and tolerability of biweekly docetaxel and gemcitabine were evaluated by clinical laboratory assessments, physical examination, and the frequency and severity of adverse events. Complete blood counts and serum biochemical assessments were performed every 2 weeks throughout the study. The severity of adverse events was graded according to the Common Toxicity Criteria Version 2.0 of the National Cancer Institute. Serious adverse events included grade 3-4 hematologic and nonhematologic toxicities. Concurrent illnesses, infections, blood product support, and antimicrobial therapies were monitored.

### 2.4. Statistical Methods

This single-institution phase II trial was planned with a Simon two-stage optimal design [22]. The primary endpoint was complete or partial response (CR + PR). We wished to distinguish these regions of the true, unknown response rate: at most 0.25 versus at least 0.45. The 2-stage design called for a maximum of 41 response-evaluable (r-e) patients, 17 in stage 1 and 24 in stage 2. The design had a type I error of 0.050 and power of 0.803.

For response and toxicity rates, Wilson type 95% confidence intervals (CI) were calculated. Response duration (RD) was measured from start of best response until relapse. Patients still in remission were censored as of the date of their last tumor assessment. Due to the small number of responders (*n* = 6), the 80% confidence level was used for the CI of RD. Time to treatment failure (TTF) was measured from registration until early discontinuation of treatment, first observation of progressive disease, or death from any cause, whichever occurred first. Patients still on treatment were censored as of the date of their last tumor assessment. Time to progression (TTP) was measured from registration until the date of documented progressive disease. Patients still progression-free were censored as of the date of their last tumor assessment. Overall survival (OS) was measured from registration to the date of death from any cause. Patients still alive were censored as of the most recent date on which they were known to be alive. Standard Kaplan-Meier estimates of the censored RD, TTF, TTP, and OS distributions were computed. Due to the small sample sizes, survival statistics (e.g., median, 6 month rate, etc.) were estimated more conservatively using linear interpolation among successive event times on the Kaplan-Meier curves [23]. 

## 3. Results

### 3.1. Patient Demographics and Disposition

Thirty-six patients were enrolled between May 2005 and May 2008. Median age was 60 years (range: 46 to 79 years) (Table 2). Ten female patients (28%) enrolled. 94% of the patients had good performance status (0-1). Patients with prior chemo-radiotherapy were 97%. The most common site of distant metastasis was lung, in 58% of the patients. The median number of cycles administered was 4 (range: 0 to 24 cycles). Three patients were registered for the study but did not receive any chemotherapy or follow up staging studies; therefore, they are considered neither r-e nor drug toxicity evaluable. In addition, 4 other patients were considered not r-e due to lack of follow-up staging studies to assess response.

### 3.2. Treatment Efficacy

Patient accrual continued to stage 2 of the study design based on the acceptable safety profile, but mainly to help define the true response rate, which appeared to be lower than predicted. Of the 29 r-e patients, 16 (55%) experienced clinical benefit (SD or disease response). Six (21%) patients achieved PR, no patients achieved CR, and the overall response rate (ORR) was 21% (95% CI: 0.10–0.38; Table 3). In an intent-to-treat analysis of all 36 patients enrolled, the ORR was 17% (95% CI: 0.08–0.32). Ten (28%) patients had SD. The median RD for the 6 responding patients was 3.2 months (80% CI: 2–6.1).

### 3.3. Hematologic Toxicity

Biweekly gemcitabine and docetaxel (GEMDOC) was generally well tolerated (Table 3). There were no treatment-related deaths. Bone marrow suppression was the main toxicity. Thirteen (39%) of the 33 treated patients had grade 3-4 anemia, and 10 (30%) patients had grade 3-4 neutropenia. Grade 3-4 thrombocytopenia occurred in only 2 (6%) patients. Only one patient had febrile neutropenia.

Twenty-five (76%) patients received all treatments without a dose reduction and twenty-seven (82%) patients had no treatment interruption.

### 3.4. Nonhematologic Toxicity

The most common grade 3-4 nonhematologic adverse event was hyponatremia, which occurred in 10 (30%) patients (Table 3). Grade 3-4 fatigue, dehydration, and dyspnea occurred in 3 (9%) patients for each toxicity. Of note, there were no reports of grade 3-4 neuropathy.

### 3.5. Time to Treatment Failure, Time to Progression, and Overall Survival

**Figure 1:**
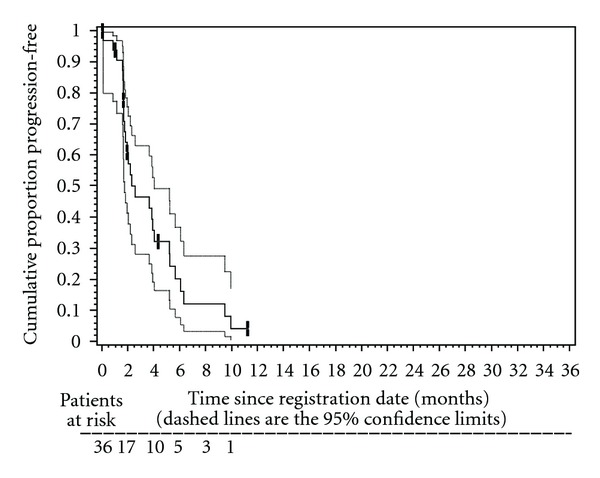
Kaplan-Meier plot of time to progression (TTP) in 36 patients treated with biweekly gemcitabine and docetaxel. Median TTP was 2.3 months (95% CI: 1.5–3.8 months). The 3-month TTP rate was 45% (95% CI: 27–63%). The 6-month TTP rate was 17% (95% CI: 1–32%).

**Figure 2:**
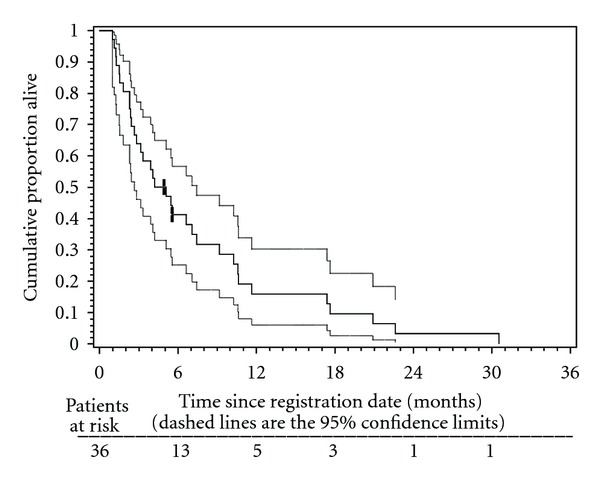
Kaplan-Meier plot of overall survival (OS) in 36 patients treated with biweekly gemcitabine and docetaxel. Median OS was 4.2 months (95% CI: 2.4–7.0 months). The 6-month OS rate was 40% (95% CI: 24–56%). The 12-month OS rate was 16% (95% CI: 3–28%).

The median TTF was 2.0 months (95% CI: 1.6 to 3.6), and the median TTP was 2.3 months (95% CI: 1.5–3.8; Figure 1). The median OS was 4.2 months (95% CI = 2.4–7.0; Figure 2).

## 4. Discussion

In this single-institution phase II trial, patients with recurrent or metastatic SCCHN were treated with the biweekly combination of gemcitabine and docetaxel (GEMDOC) after the failure of at least one prior chemotherapy regimen. Of the 29 response evaluable patients only 6 achieved PR and there was no CR observed. It was concluded that the sample response rate among the r-e patients (6/29 = 21%) better supported the null hypothesis that the true, unknown response rate was at most 25%. However, the clinical benefit (PR + SD) rate was 55%. One proposed reason for the low response rate is the type of recurrent disease treated, as the majority of patients included in this trail had previous one to two lines of chemotherapy or concurrent chemo-irradiation with unresectable relapses in the radiation field. The study design excluded metastatic-chemotherapy-naïve patients. Median response duration in the 6 responders was 3.2 months. Median time to treatment failure was 2.0 months, with median survival of 4.2 months (Figure 2).

Biweekly treatment with gemcitabine and docetaxel was generally well tolerated. The median number of cycles administered was 4. Bone marrow suppression was the main toxicity. Ten (30%) patients had grade 3-4 neutropenia, 25 (76%) patients received all treatments without a dose reduction, and 27 (82%) patients had no treatment interruption. This trial explored a new potential treatment option in a bad prognosis group of patients with metastatic or recurrent SCCHN.

Although the studied treatment regimen was safe, the response rate was lower than predicted and the study was terminated. Docetaxel is a well-established agent in the treatment of SCCHN [6–8, 24], and probably there was more added efficacy by combing it with gemcitabine.

Recent data in recurrent or metastatic SCCHN showed better treatment efficacy driven from combination chemotherapy with cisplatin, 5-fluorouracil, and cetuximab [25]. Future clinical trials in this setting should be based on the use of doublets, either platinum based or taxane based, with a targeted agent. Given the good safety profile and the overall clinical benefit of the biweekly GEMDOC regimen, further investigation of this combination with the addition of targeted agents may lead to better efficacy results.

## Figures and Tables

**Table 1:** Phase II studies of biweekly gemcitabine and docetaxel (GEMDOC).

Author	*N*	Tumor type	Treatment regimen	Toxicity
Galetta et al. [11]	45	NSCLC	Gemcitabine 2000 mg/m^2^ q 2 weeks and docetaxel 50 mg/m^2^	Grade 3 and 4 neutropenia 23%, Grade 3 and 4 alopecia 34%
Pelegri et al. [12]	36	Breast cancer	Gemcitabine 2500 mg/m^2^ q 2 weeks and docetaxel 65 mg/m^2^	Grade 3 and 4 neutropenia 45%
Syrigos et al. [13]	25	NSCLC	Gemcitabine 1000 mg/m^2^ q 2 weeks and docetaxel 80 mg/m^2^	Anemia 16%, neutropenia 20%, febrile neutropenia 10%, diarrhea 24%, and asthenia 64%
Shepard et al. [14]	32	Pancreatic	Gemcitabine 2000 mg/m^2^ q 2 weeks and docetaxel 75 mg/m^2^	No grade 4 toxicity Grade 3 neutropenia 31%

**Table 2:** Baseline characteristics and clinical variables for all 36 patients.

Characteristic	*N* (%)
Age (years)	
Median (range)	60 (46–79)
Sex	
Female	10 (28%)
Male	26 (72%)
Race	
Caucasian	22 (61%)
African-American	12 (33%)
Other	2 (5%)
Performance status (ECOG)	
0	8 (22%)
1	26 (72%)
2	2 (6%)
Prior chemo-radiotherapy	
No	1 (3%)
Yes	35 (97%)
Sites of metastasis	
Lung	21 (58%)
Liver	2 (6%)
Other	6 (17%)
Treatment cycles administered	
Median (range)	4 (0–24)

Percentages may not sum up to 100 due to rounding.

Multiple sites of metastasis may have occurred in the same patient.

**Table 3:** Grade 3-4 toxicity summary statistics for all 33 treated patients.

Grade 3-4 toxicity	*N*	Events	Point estimate	95% confidence interval	
Anemia	33	13	39%	25%	56%
Neutropenia	33	10	30%	17%	47%
Hyponatremia	33	10	30%	17%	47%
Dehydration	33	3	9%	3%	24%
Fatigue	33	3	9%	3%	24%
Dyspnea	33	3	9%	3%	24%
Pneumonia	33	3	9%	3%	24%
Thrombocytopenia	33	2	6%	2%	20%
Febrile neutropenia	33	1	3%	1%	15%
Tachycardia	33	1	3%	1%	15%
Syncope	33	1	3%	1%	15%
Fluid retention	33	1	3%	1%	15%
Mucositis	33	1	3%	1%	15%
Hyperglycemia	33	1	3%	1%	15%
Constipation	33	1	3%	1%	15%
Anorexia	33	1	3%	1%	15%
Vomiting	33	1	3%	1%	15%
Other nonhematologic toxicity	33	1	3%	1%	15%
